# Analyses of the differentiation potential of satellite cells from *myoD*^-/-^, *mdx*, and *PMP22 *C22 mice

**DOI:** 10.1186/1471-2474-6-15

**Published:** 2005-03-11

**Authors:** Marion M Schuierer, Christopher J Mann, Heidi Bildsoe, Clare Huxley, Simon M Hughes

**Affiliations:** 1 Insitute of Pathology, Medical School of the University of Regensburg, Franz-Josef-Strauss-Allee 11, 93053 Regensburg, Germany; 2 Division of Biomedical Sciences, and Clinical Sciences Centre, Imperial College School of Science, Technology and Medicine, London, UK; 3 MRC Centre for Developmental Neurobiology and Randall Division for Cell and Molecular Biophysics, Guy's Campus, King's College, London, UK

## Abstract

**Background:**

Sporadic and sometimes contradictory studies have indicated changes in satellite cell behaviour associated with the progressive nature of human Duchenne muscular dystrophy (DMD). Satellite cell proliferation and number are reportedly altered in DMD and the *mdx *mouse model. We recently found that satellite cells in MSV*ski *transgenic mice, a muscle hypertrophy model showing progressive muscle degeneration, display a severe ageing-related differentiation defect *in vitro*. We tested the hypothesis that similar changes contribute to the gradual loss of muscle function with age in *mdx *and *PMP22 *mice, a model of human motor and sensory neuropathy type 1A (HMSN1A).

**Methods:**

Single extensor digitorum longus muscle fibres were cultured from *mdx *and *PMP22 *mice and age- and genetic background-matched controls. Mice at several ages were compared with regard to the differentiation of satellite cells, assayed as the proportion of desmin-expressing cells that accumulated sarcomeric myosin heavy chain.

**Results:**

Satellite cells of 2 month, 6 month, and 12 month old *mdx *mice were capable of differentiating to a similar extent to age-matched wild type control animals in an *in vitro *proliferation/differentiation model. Strikingly, differentiation efficiency in individual 6 month and 12 month old *mdx *animals varies to a much higher extent than in age-matched controls, younger *mdx *animals, or *PMP22 *mice. In contrast, differentiation of myoblasts from all *myoD *null mice assayed was severely impaired in this assay system. The defect in satellite cell differentiation that occurs in some *mdx *animals arises from a delay in differentiation that is not overcome by IGF-1 treatment at any phase of cultivation.

**Conclusion:**

Overall, a defect in satellite cell differentiation above that arising through normal ageing does not occur in *mdx *or *PMP22 *mouse models of human disease. Nonetheless, the impaired differentiation of satellite cells from some *mdx *animals suggests that additional factors, environmental or epigenetic, may lead to deteriorating muscle repair through poor differentiation of satellite cells in genetically predisposed individuals.

## Background

Like many muscle diseases, Duchenne muscular dystrophy (DMD) is characterised by a gradual loss of muscle function with age. Patients are initially ambulatory and have mild muscle pathology, despite ongoing degeneration and repair. In later stages of DMD, patients experience progressively more severe muscular changes, accompanied by loss of function, physical dependency, and ultimately, death [1].

DMD patients generally lack the cytoskeletal protein dystrophin, a member of the spectrin-like superfamily of actin binding proteins. Functional dystrophin is localised to the inner face of the sarcolemma and binds to cytoskeletal F-actin and transmembrane beta-dystroglycan as part of multiprotein complex that mediates signalling between the cytoskeleton and the extracellular matrix. The consequences of lack of dystrophin appear to be an enhanced susceptibility to fibre damage and possibly poor signalling between fibres and their environment. A milder form of dystrophin deficiency in humans is the Becker-Kiener type of muscular dystrophy (BMD). Here, dystrophin is not completely absent, but mutations lead to a quantitatively and/or qualitatively reduced gene product which does not accomplish its full function. The onset of BMD is generally later than DMD. In childhood, symptoms are usually very mild and muscle weakness becomes more evident only in the teens or twenties. BMD is non-lethal and patients often achieve normal life span, although the disease can progress in later life [2].

To date, it is not clear what controls disease progression in either DMD or BMD and no consensus has been reached in the literature. The progressive loss of muscle in DMD and other muscle disorders could be due to a sustained or increasing rate of degeneration above the rate of repair, or a progressive decline in the ability to regenerate the muscle. Some pathological changes appear to be similar to those observed in healthily ageing people, yet premature and exacerbated. A popular, yet challenged view is that in DMD, disease progression is attributable to accumulating deficiencies in the ability of satellite cells resident within the muscle to mediate regeneration and/or their own replacement [3]. Satellite cell deficiencies could arise because of the excessive demands on repair mechanisms necessitated by the continuous degeneration of unstable muscle that does not express dystrophin. Indeed, symptoms of rapid early turnover of muscle fibre material and cells are apparent before birth in DMD patients [4], yet serious functional deficits arise only late in the first decade.

A commonly used model for studies of DMD is the *mdx *mouse. These animals, like human patients, show delayed onset of debilitating muscle degeneration. Although a transient burst of frank degeneration occurs in *mdx *mice during the period of muscle growth around the third postnatal week, degeneration leading to debility only occurs late in life, primarily in specific muscles, such as the diaphragm [5]. In *mdx *mice, as in human DMD patients, disease is caused by the absence of functional dystrophin, owing to a nonsense mutation in exon 23. The relatively mild phenotype of *mdx *mice can, in part, be attributed to the compensatory function of the dystrophin-related protein utrophin, which is highly upregulated in regenerating muscle fibres in adult *mdx *mutants [6]. Over time, enhanced muscle turnover and satellite cell numbers are also seen in *mdx *mice [7].

Other muscle diseases also show variable clinical progression. Human motor and sensory neuropathy type 1A (HMSN1A, also known as Charcot-Marie-Tooth disease type 1A, CMT1A) is a dominantly inherited demyelinating disorder of the peripheral nervous system. It is most frequently caused by over-expression of the *PMP22 *gene due to duplication of a 1.5-Mb region on chromosome 17, but it can also result from point mutations in the *PMP22 *gene [8-12]. The affected individuals typically have distal muscle weakness and atrophy often associated with mild to moderate sensory loss, depressed tendon reflexes, and high-arched feet. Individuals with HMSN1A experience slowly progressive weakness and atrophy of distal muscles in the feet and/or hands. Disease progression is variable for unknown reasons.

PMP22 C22 transgenic mice which were modified to harbour seven copies of the human *PMP22 *gene demonstrate developmental delays in myelination, decreased numbers of myelinated fibres, and abnormally thin myelin similar to HMSN1A [13]. Being a neuropathy, PMP22 C22 mice can be used as reference animals that display a muscle phenotype without harbouring intrinsic muscle defects.

In a recent study, we found that satellite cells of MSV*ski *transgenic mice display a differentiation defect compared to wildtype control animals and that this defect is exacerbated in ageing animals [14]. Like *mdx *mice, hypertrophic MSV*ski *transgenic mouse muscles have muscle degeneration that is initially efficiently repaired, but which eventually shows defective regeneration and frank muscle defects. In the present paper, we investigate the differentiation potential of satellite cells of single muscle fibres from the hypertrophic *mdx *and *PMP22 *mouse models and corresponding wildtype control animals in order to clarify whether ageing-related change in differentiation potential of satellite cells might influence disease progression.

## Methods

### Mouse strains

*mdx *mice were obtained from a colony in the lab of T. Partridge (Imperial College, London, UK) and are similar to JAX C57BL/10ScSn-Dmd^*mdx*^/J. Control mice were obtained from JAX and were C57BL/10ScSn. MyoD^m1 ^null mice were as reported [15]. Mice were killed at ages of 6 to 8 weeks (referred to as 2 months), 22 to 25 weeks (referred to as 6 months), or 44 to 55 weeks (referred to as 12 months). Mice of the HMSN1A model PMP22 C22 and the corresponding age matched litter mate controls were received from the lab of C. Huxley at ages of 2 to 3 months (referred to as 2 months), 10 to 12 months (referred to as 11 months), and 15 months. Mice were kept in plastic cages with wire mesh lids in a 12:12-h light-dark cycle and fed *ad libitum*. Both sexes were used for each experimental time point to test for sex specific effects, although none were observed, and at least 4 mice of each genotype were used at each age. All animal experiments were carried out in accordance with the local ethics committee and UK Home Office approval.

### Single fibre preparation

Single fibres from mouse EDL or soleus muscle were isolated and cultured in order to obtain satellite cells as described recently [14]. Briefly, muscle tissue was dissected from mice of appropriate age and genotype in a manner that minimised injury, stretch, or other stress factors on the fibres. Connective tissue was removed by incubation in DMEM (Gibco, Paisley, UK) with 0.2% collagenase type I at 35°C for 1 h. Fibres were liberated by trituration in DMEM medium with Pasteur pipettes of different pore sizes. Fibres were fixed in 4% PFA, or placed in isolated wells of 8-well Permanox™ chamber slides (NalgeNunc International, Rochester, USA), coated with Matrigel (1 mg/ml in DMEM, BD Biosciences, Oxford, UK) for satellite cell cultivation and immunocytochemistry.

### Satellite cell cultivation

Single fibres were allowed to adhere to the Matrigel matrix (3–5 mins), before adding 300 μl of plating medium (10% horse serum, HS (Gibco), 0.5% Chick Embryo Extract, CEE (Sera Laboratories International Ltd., Crawley, UK), in DMEM with 1% streptomycin/penicillin and 2% L-glutamine) and incubation in a humidified environment at 37°C and 5% CO_2_. After 3 days incubation to allow satellite cells to migrate off the fibre onto the Matrigel substrate, fibres were removed from the chambers and medium was replaced by proliferation medium (PM; 20% fetal calf serum, FCS, 10% HS, 2% CEE in DMEM). After further two days, PM was replaced by differentiation medium (DM ; 2% FCS in DMEM) and cells were allowed to differentiate for 2 or 5 days. Three days after plating and again after two days in proliferation medium, the total number of cells was analysed for each single fibre culture. To check for the influence of IGF-1 treatment on differentiation efficiency of satellite cells, PM, DM, or both were supplemented with 100 ng/ml of recombinant R^3^-IGF-1 (Sigma, Deisenhofen, Germany).

### Immunocytochemistry

Slides were rinsed in PBS, fixed in cold methanol, blocked in 5% horse serum in PBS, and incubated with mAb against desmin (1/500, clone no. DE-U-10, IgG1; Sigma) and MyHC (1/10, A4.1025, IgG2a; [16]). Primary antibodies were successively detected with rat anti-mouse IgG1 (1:1,000; Serotec, Oxford, UK), FITC-conjugated goat anti-mouse IgG2a (1:100; Serotec, Oxford, UK), and Cy3-conjugated donkey anti-rat IgG (1:100; Jackson, USA). In the last antibody incubation, the DNA dye DAPI was added. Cells were washed, re-fixed in cold methanol and mounted with antifading agent. Cell differentiation was analysed by monitoring 10 randomly-selected fields of view equivalent to a total area of 5.45 mm^2 ^of each chamber, representing approximately 15% of the area of each chamber. Differentiation efficiency (labelled 'Myoblast differentiation') was determined as the ratio of nuclei in myosin heavy chain positive (MyHC^+^) myocytes and/or myotubes divided by the total number of nuclei in desmin-expressing cells (desmin^+ ^cells). Data presented reflect means and standard deviation of fibres from each of ≥ 4 animals unless otherwise stated. Cultures from ≥ 30 fibres were analysed in each case.

## Results

### A sensitive assay for satellite cell differentiation

**Figure 1:**
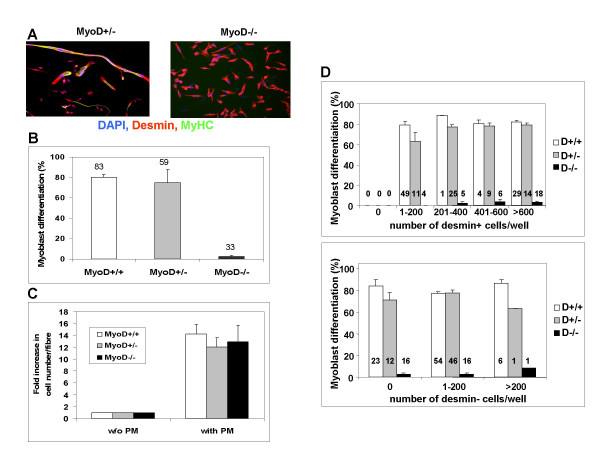
**A method to quantitate satellite cell differentiation. **Single fibres from EDL muscles of 2 month old mice were isolated and cultured for five days, switched to differentiation medium for two days and the fraction of desmin-positive (desmin^+^) cells expressing myosin heavy chain determined by immunofluorescence microscopy. **A: **Satellite cell cultures from *myoD *heterozygous (*myoD*^+/-^) and homozygous null (*myoD*^-/-^) animals. Essentially all cells (blue nuclei: DAPI) express desmin (red: Cy3) in both genotypes, but note the lack of multinucleate myotubes and cells expressing MyHC (green: FITC) in the *myoD*^-/-^. **B-D: **Quantification of satellite cell-derived myoblast growth and differentiation by genotype. Error bar = SD. Numbers above columns indicate number of wells counted (1 fibre/well) from 4 animals of *myoD*^+/+^, *myoD*^+/-^, and *myoD*^-/- ^genotype, respectively. **B: **Differentiation of *myoD*^-/- ^myoblasts is poor compared to littermates. **C: **Increase in cell number after the switch from plating to proliferation medium is independent from the genotype of the animals. *myoD*^+/+^, *myoD*^+/- ^and *myoD*^-/- ^cells proliferate to the same extent. **D: **The fraction of differentiated myogenic cells is unaffected by either the number of myogenic desmin^+ ^cells or the number of non-myogenic desmin^- ^cells in each well. In all genotypes (*myoD*^+/+^, *myoD*^+/-^, and *myoD*^-/-^) all fibres yielded myogenic cells.

Mice wild type or carrying one or two null alleles at the *myoD *locus were used to establish an *in vitro *culture system to measure the differentiation potential of satellite cells in culture. In agreement with published data [17], satellite cells from single EDL muscle fibres from wildtype or heterozygous *myoD*^+/- ^animals showed high levels of differentiation, as assayed by the fraction of desmin^+ ^myogenic cells that express MyHC, whereas satellite cells from *myoD *deficient animals often failed to differentiate (Fig. 1A,B). Satellite cells of *myoD *wildtype mice (*myoD*^+/+^) displayed efficient differentiation (80% ± 3) within 2 days of growth factor removal. Comparable numbers of satellite cells from *myoD*^+/- ^EDL muscles differentiated terminally (75% ± 14), whereas cells obtained from *myoD*^-/- ^muscle fibres almost completely failed to differentiate (3% ± 1). Thus, single fibre cultures can efficiently quantify myoblast differentiation.

We checked that the myogenic cell yield and proliferation of satellite cells did not differ between *myoD*^+/+^, *myoD*^+/-^, and *myoD*^-/- ^fibres (Fig. 1C). During the 2 days in PM around a 12- to 14-fold increase in cell numbers occurred, indicating an approximately 12 hour doubling time, independent of genotype.

The presence of non-myogenic cells and/or total cell density might affect myogenic differentiation efficiency. Therefore, we used desmin expression to discriminate between myogenic and non-myogenic cells. Differentiation efficiency in wells with different numbers of desmin positive (desmin^+^) or desmin negative (desmin^-^) cells for all cultures of *myoD*^+/+^, *myoD*^+/^, and *myoD*^-/- ^fibres was compared (Fig. 1D). No effect of desmin^- ^cells in the cultures was detected on the differentiation potential of the myogenic cells. On average, there were less than 11% non-myogenic cells in any cultures from mice of any genotype. Similarly, differentiation efficiency did not correlate with the density of either total cells or desmin^+ ^cells; satellite cells are capable of differentiating (expressing MyHC) independently of cell fusion. Therefore, in this culture system, density of neither myogenic cells nor non-myogenic cells has a detectable influence on differentiation efficiency.

### Differentiation potential of satellite cells from PMP22 transgenic mice

**Figure 2:**
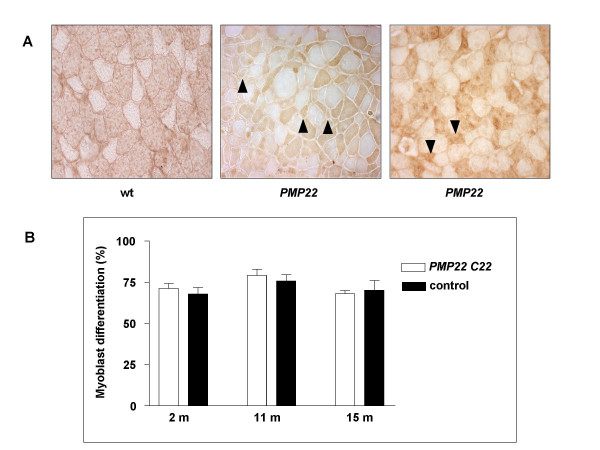
**Differentiation of satellite cells of the HMSN1A mouse model PMP22 C22 at three ages. A. **Variation in soleus muscle cryosections reacted for slow MyHC reveals variable pathology of individual *PMP22 C22 *mice. Note fibre atrophy (arrowheads) and large, hypertrophied fibres in the middle compared to the right panel. **B. **Satellite cells from single fibre culture of *PMP22 C22 *and littermate controls were analysed for cell yield, proliferation and terminal differentiation. No significant differences were observed.

To test whether reduced muscle motility, altered innervation, or fibre atrophy/regrowth might trigger satellite cell changes in some individuals, we examined *PMP22 *C22 transgenic mice that all show signs of disease, but have a heterogeneous progression. Mouse models have demonstrated that excess PMP22 protein in Schwann cells leads to repeated focal demyelination lesions in peripheral nerves, decreased conduction velocity, and a muscle histology characteristic of partial denervation, just as in HMSN1A [13]. Histological analysis of sections of soleus muscles revealed that in some individual animals signs of dramatic fibre atrophy and/or regrowth are present (Fig. 2A). In addition, significant changes in fibre type occur, as revealed by MyHC isoform expression. In other individuals, however, the alteration in muscle fibre size profile and type was less marked (data not shown). To clarify whether phenotypic variation is reflected in diversity of satellite cell behaviour, we analysed satellite cells of *PMP22 *C22 transgenic mice at several ages. However, no significant variation in satellite cell differentiation efficiency was observed at any age examined (Fig. 2B). Again, no influence of myogenic or non-myogenic cell density on differentiation was observed (data not shown). Little inter-animal variation was observed, unlike the case of *mdx *animals (see below). Thus, *PMP22 *mice show no loss of satellite cell function.

### Differentiation potential of satellite cells of *mdx *mice

**Figure 3:**
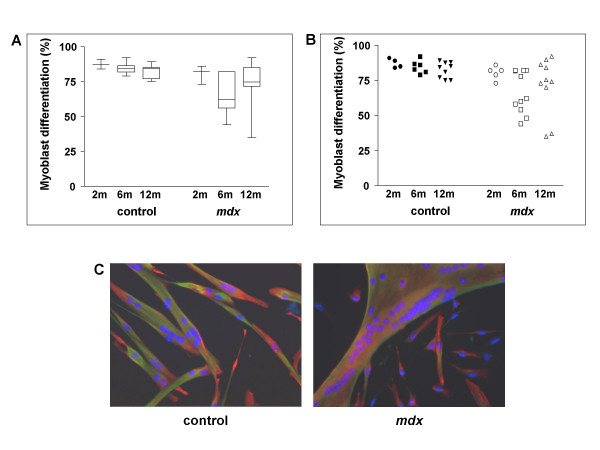
**Differentiation of satellite cells of control and *mdx *mice of different ages *in vitro. *A. **Box and whisker plot: The box extends from the 25th percentile to the 75th percentile between all animals of one age, with a horizontal line at the median (50th percentile). Whiskers show the range of the data. **B. **Mean differentiation for each individual animal shows heterogeneity between *mdx *animals. Individuals showing poor differentiation (8 in total) are significantly more common in *mdx *(X^2^, P = 0.0076). **C. **Satellite cell cultures from single fibres of 12 month old control C57BL/10ScSn and *mdx *animals showing good differentiation in each case. Blue nuclei: DAPI; desmin: red, Cy3; MyHC: green, FITC in both genotypes.

To test whether satellite cells from EDL muscles of *mdx *mice display a defect in differentiation, we compared single fibre cultures of *mdx *with age-matched control animals, on the same genetic background, C57BL/10. In fibre cultures from 2 month old control and *mdx *animals differentiation was 87% ± 3 and 80% ± 5, respectively (Fig. 3A–C). Thus, the early phase of significant degeneration/regeneration in *mdx *mice led to no detectable change in satellite cell differentiation.

We have observed decline in differentiation capacity as satellite cells age in regenerating muscle [14]. In the present study, satellite cells from control C57BL/10 mice yield high levels of differentiation at all ages examined with only a slight and not significant decrease with age (87% ± 3, 84% ± 5, and 82% ± 6 in fibres from 2, 6, and 12 month old mice, respectively; Fig. 3A). Similar experiments were performed for *mdx *animals of corresponding ages. Across all fibres from all *mdx *animals, no significant difference in differentiation in comparison to wildtype animals was observed. Mean differentiation efficiency was 80% (± 5) at 2 months old, 66% (± 15) at 6 months old, and 72% (± 20) at 12 months old *mdx *animals. However, as indicated by the high standard deviation, the variance in differentiation was extremely high in older animals.

We investigated the reason for the higher variance in differentiation of 6 and 12 month *mdx *cultures. Whereas numbers of cells per well and desmin^+ ^or desmin^- ^cell density showed no apparent contribution to variance, animal to animal differences were striking. Many individual *mdx *mice yielded satellite cells capable of differentiating as well as age-matched controls. However, some individual animals yielded particularly poorly differentiating cells (Fig. 3B). This variation could not be accounted for by day to day variation in culture conditions because age-matched *mdx *and wild type animals were always prepared in parallel. There was no indication of correlation with sex, health status, housing conditions, or season, although numbers of animals were too low to eliminate any of these variables with high confidence.

### Differentiation defect of affected *mdx *satellite cells can be overcome by prolonged differentiation time

**Figure 4:**
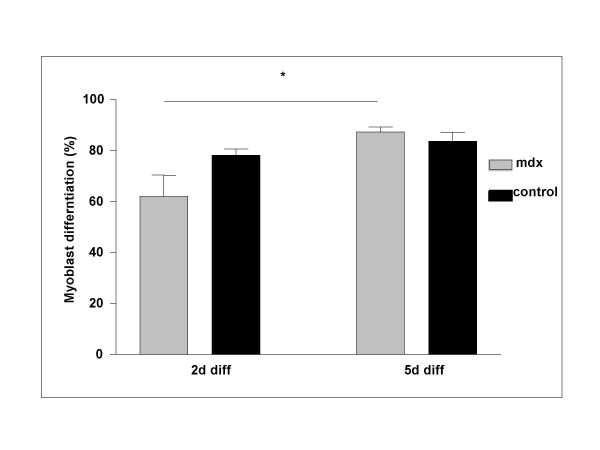
**Prolongation of differentiation rescues the satellite cell differentiation defect. **Satellite cells of 6 months old *mdx *or control animals were plated and allowed to proliferate as described in Materials and Methods and then cultivated either for 2 days (2d diff) or for 5 days (5d diff) in differentiation medium. Prolongation of the differentiation period to 5 days enhances differentiation efficiency (* P < 0.0028) of affected *mdx *satellite cells to the level observed in controls.

Poor satellite cell differentiation in some older *mdx *mice could reflect a permanent block or simply a delay. We therefore examined differentiation rate in satellite cells from older animals that showed poor differentiation. Single fibre cultures were allowed to differentiate in low-serum medium for 5 days (5d diff) instead of the normal 2 days (2d diff) employed in all other experiments. Prolonged differentiation did not significantly enhance differentiation of control cultures (78% ± 6 and 83% ± 5 after 2 and 5 days, respectively). However, a prolonged differentiation period did rescue differentiation of *mdx *cells to levels indistinguishable from control 5 day cultures (Fig. 4). A significant increase in differentiation capacity from 63% ± 6 to 87% ± 2 in *mdx *mice was observed. We conclude from this result that satellite cells from some *mdx *mice differentiate less efficiently in our experimental system as reflected by a slower rate of MyHC accumulation.

### IGF-1 treatment does not influence the differentiation of satellite cells *in vitro*

**Figure 5:**
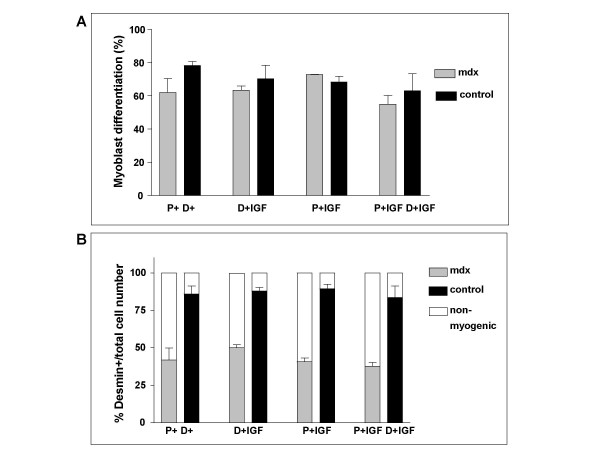
**IGF-1 treatment fails to rescue differentiation of satellite cells in culture. **Satellite cells from control or *mdx *animals at the age of 6 months were treated with IGF-1 (100 ng/ml medium) during either proliferation (P+IGF), differentiation (D+IGF), both (P+IGF D+IGF), or neither (P+D+, control). **A**. Differentiation was unaltered by IGF-1 addition. **B**. Non-myogenic cells are more abundant in *mdx *than control single fibre cultures.

IGF-1 signals have been shown to enhance both proliferation and differentiation of satellite cells [18]. To begin to define the nature of the emerging satellite cell defect in muscles of some *mdx *mice, we exposed satellite cells from 6 month old control or poorly-differentiating *mdx *animals to IGF-1 (100 ng/ml) either in the proliferation phase, the differentiation phase or both (P+IGF, D+IGF, P+IGF D+IGF, respectively; Fig. 5). Controls were cultivated for the same time periods in the appropriate medium without IGF-1 (P+ D+). No significant effect of the IGF-1 treatment on the terminal differentiation of satellite cells was observed in any of the culture stages (Fig. 5A). Thus, IGF-1 level is not rate limiting for differentiation of *mdx *cultures that show reduced differentiation rate. Neither can IGF-1 promote differentiation of the undifferentiated ~20% of desmin^+ ^cells in control cultures.

As controls, we checked that the proportion of myogenic, desmin^+ ^cells in the cultures was not altered significantly by IGF-1 treatment in relation to total cell number in either control or *mdx*. However, the fraction of non-myogenic cells was over three times greater in *mdx *(58% ± 6) than in controls (16% +/- 5), independent of IGF-1 treatment (Fig. 5B). As found in the experiments above, despite this difference in non-myogenic cell numbers, differentiation efficiency was comparable between *mdx *and controls (Fig. 5A).

## Discussion

Satellite cells are normally quiescent cells that reside under the basal lamina of muscle fibres. Under conditions of growth and repair, satellite cells become activated and begin a coordinated myogenic program: they initially proliferate and express a range of myogenic genes, including desmin, before aligning and fusing to form terminally differentiated multinucleate syncitia that organise and express the contractile apparatus, which includes the MyHC proteins. We have shown recently that, in *MSVski *transgenic mice, satellite cell differentiation *in vitro *was influenced by the age of the study animals and showed a progressive decline with age [14]. This effect was much less obvious in wildtype control animals. Therefore, we concluded that a satellite cell differentiation defect developed in *MSVski *mice. We speculated that the defect might be caused by continual degeneration/repair apparent in hypertrophic *MSVski *mice and that the changes in satellite cells might underlie the worsening of the muscle pathological profile with age. The *mdx *mouse model of human DMD displays a hypertrophy phenotype in the skeletal musculature reminiscent of *MSVski *mice. Similarly, DMD patients initially show a mild phenotype that gradually progresses throughout the lifespan, manifesting as muscle loss and fibrosis which culminates in death from respiratory or cardiac failure. As the progression of DMD is not understood and because of similarities between DMD/*mdx *pathology and the MSV*ski *animal phenotype we hypothesised that progressive defect in the differentiation potential of satellite cells might contribute to the pathologic mechanism of the debilitating human disease.

We find that satellite-derived cells from ageing *mdx *mice are, in general, capable of differentiating to the same degree as satellite-derived cells from control animals. We did not assess satellite-derived cells that remained physically juxtaposed to the explanted fibre because such cells could display different behaviour(s) due to variations in the fibre integrity or its surrounding matrix. We chose to measure differentiation as the fraction of desmin-expressing cells co-expressing MyHC rather than fusion for several reasons. Firstly, without using assays that detect syncitia [19], there is no way of clearly ascertaining whether two cells are truly fused or closely apposed. Secondly, mononucleate cells are capable of terminally differentiating and expressing MyHC; such cells would be missed if assessing fusion. Although we can not eliminate the possibility that the *mdx *condition leads to an alteration in the cells that express desmin, our study provides evidence against this view. First, yields of desmin^+ ^cells are similar between *mdx *and control. Second, the proliferation rate of desmin^+ ^cells appears similar between *mdx *and control. Third, and as discussed further below, although numbers of desmin^- ^cells are increased in *mdx *cultures the increase is similar both before and after the differentiation phase suggesting that interconversion of desmin^- ^and desmin^+ ^cells is not a significant factor in our experiments. Overall, it is unlikely that a defect in differentiation of satellite-derived cells is a major contributor to disease progression in *mdx *mice.

Despite this lack of significant change in overall differentiation capacity, satellite-derived cells from some individual older *mdx *animals displayed lower differentiation efficiencies than those from other *mdx *animals of the same age. Age- and background genotype-matched control mice, young *mdx *mice, and mice with severe muscle weakness due to the *PMP22 *transgene did not show this variation. We were unable to correlate this effect to any factor analysed. All animals were held under similar conditions. We consciously used both genders and analysed data in combination of both sexes and separately. We conducted additional experiments using increased or decreased collagenase type I incubation times as well as a more severe collagenase type II digestion to determine if variations in satellite cell activation or yield, basal lamina digestion, or fibre damage could affect differentiation potential, but found no differences (data not shown). Similarly, we performed dilution cloning of satellite cells to assess the effects of proliferation rate on satellite cells and whereas we observed heterogeneity of proliferation rates amongst satellite cells grown from single cells, we again found no ultimate difference in differentiation efficiency (data not shown). For those reasons we can not explain the inter-animal variation of *mdx *results by differences in the experimental design or genetic background of the animal. We speculate that uncontrolled environmental effects or epigenetic factors affecting other genes in the *mdx *background explain the variation. It is striking that fibres yielding poorly-differentiating cells are numerous in affected individuals, but nevertheless, some fibres yield cells differentiating as well in controls. This emphasises that relatively heritable heterogeneity in myogenic cells must exist in *mdx *mice and demands elucidation. Moreover, we can not eliminate the possibility that the *mdx *individuals showing poor differentiation in our assay would have undergone a worse progression of disease in later life. Additionally, we cannot exclude the possibility that very subtle differences in differentiation behaviour were not detected in our assay system as we have utilized matrigel, a matrix in which growth factors are abundant. Thus, small variations might have been masked that only would be detectable at the application of collagen or gelatine matrices.

As shown by others [20] and in this report, non-myogenic cells, probably fibroblasts, can be obtained from single fibre cultures and are more abundant in *mdx *samples compared with C57BL/10 controls. These cells probably reside on the fibre surface and migrate away from the fibre onto the substrate as do satellite-derived cells. *In vivo*, these cells may mediate the fibrotic response to fibre degeneration and could potentially secrete factors such as TGF-β that have been shown to interfere with satellite cell differentiation [21]. We analysed the proportion of non-myogenic cells in the cultures and whether they influenced the efficiency of differentiation of myogenic cells. We were unable to find a correlation between the contamination of the satellite cell culture with desmin^- ^non-myogenic cells and the differentiation efficiency of the myogenic cells in the same culture well. This confirms what we have observed in wild type, *MSVski*, *PMP22*, and *myoD *null situations [14]. There was also no difference in desmin^- ^cell levels between mice showing poor or normal differentiation. Thus, at least in *mdx *animals up to one year of age, no correlation of fibrosis with poor myoblast differentiation is apparent.

### Differentiation is delayed, not inhibited in some *mdx *mice

We found that satellite-derived cells from *mdx *mice showing poor differentiation after two days differentiation, recovered and differentiated as well as controls after three further days in differentiation conditions. No morphological differences in the nature of the differentiated cells were detected at this stage. Thus, the reduction in differentiation observed in some *mdx *animals is most simply explained as a reduced rate of differentiation. If such a decrease in differentiation rate occurs *in vivo*, it could have serious consequences for muscle repair, which may require rapid satellite cell mobilisation and can occur within a few days [22].

IGF-1 treatment has been shown to enhance the efficiency of differentiation of satellite-derived myoblasts and this has been suggested to mediate an autocrine loop triggered by myogenin expression [23]. However, IGF-1 treatment of poorly-differentiating *mdx *satellite-derived cells did not enhance their differentiation rate. Moreover, although it has been reported that IGF-treatment of wildtype myogenic cells leads to enhanced proliferation [24,25], in our experimental conditions using recombinant R^3 ^IGF-I we were not able to confirm those findings.

### Functional impairment in HMSN1A model mice is not accompanied by satellite cell changes

A rodent model for the progressive human neuropathy HMSN1A is the *PMP22 C22 *transgenic mouse that harbours seven copies of the human *PMP22 *gene in its genome. These animals manifest symptoms similar to human HMSN1A patients including demyelination of Schwann cells and, with later age, progressive skeletal muscle weakness caused by poor innervation [13]. Examination of postural soleus muscle from such mice revealed a heterogeneous progression of muscle pathology. Some animals showed severe signs of atrophic fibres and changes in fibre type proportion, probably caused by altered electrical activity consequent to slowed nerve conduction. Other individuals, which also showed poor movement, had relatively healthy muscle histology. A cohort of affected *PMP22 *C22 mice with altered gait were analysed by single fibre culture and revealed no changes in number, proliferation, or differentiation of desmin^+ ^cells compared to age- and genetic background-matched controls. Thus, the HMSN1A model mice show a strong phenotype which seems to involve muscle fibre atrophy after demyelination of its innervating motorneuron followed by satellite cell recruitment in the regrowth phase after myelination is restored. However, these processes have no observable effect on mononucleate cells in single fibre cultures.

### Differentiation defect in satellite cells lacking *myoD*

There has been controversy surrounding the role of the myogenic transcription factor MyoD in satellite cell differentiation. Early reports suggested the *myoD *null muscle regenerated poorly and that myoblasts from young *myoD *null mice differentiated poorly *in vitro *[17,26]. However, a recent study showed the *myoD *null satellite cells can differentiate efficiently under some circumstances [27]. In our single EDL muscle fibre cultures, satellite-derived myoblasts from wild type or heterozygous *myoD*^+/- ^mice differentiate efficiently. In contrast, few desmin-expressing satellite-derived cells from *myoD *deficient mice were able express MyHC within two days. Given the rapid repair of entire muscles within several days of toxin-induced injury [22], the differentiation delay in *myoD *null would have severe consequences in wild populations if a similar delay occurred in any *in vivo *setting.

An issue not addressed directly by our study is the relationship of the assayed population of desmin-expressing cells from wild type to that from *myoD *null animals. For example, lack of *myoD *might cause a change in the numbers of cells expressing desmin. We think this unlikely because the yield of desmin^+ ^cells, and the ratio of desmin^+ ^to desmin^- ^cells, were unaltered in our single fibre cultures, irrespective of *myoD *genotype. Whatever the case, our experiment shows that lack of *myoD *leads to substantial reduction in the capacity of muscle fibre-associated migratory proliferative cells to undergo terminal differentiation into myotubes.

## Conclusion

As there appears to be no distinct differentiation defect in the satellite cells of *mdx *mice, at least by our assay, the cause of progressive muscle loss in the diseased state remains unclear. The differentiation potential of satellite cells has generated contradictory results in several early studies, where differentiation was measured as myoblast fusion in primary cultures (reviewed in [28,29]). One study concluded no differentiation defect [4], whereas Jasmin and colleagues found a reduced differentiative capacity in myoblasts derived from DMD patients [30]. A very recent publication by Schaefer et al. also revealed heterogeneity in the number of satellite cells between individual mdx and C57 animals. The authors observed that this heterogeneity did not correlate with age, gender, or degree of degeneration, but possibly reflected additional genetic factors that influence the maintenance of the satellite cell pool [31].

Altered myoblast number appears not to explain disease progression. Some studies find an increase in the number of satellite cells associated with DMD/*mdx *muscle fibres ([32-35] and our unpublished observations), although others observe little change [7]. Thus loss of available cells for repair is unlikely to cause disease progression. Despite reports of increased cell death in DMD/*mdx *myoblasts [36-38], we found no evidence of differential apoptosis between control and *mdx*. It has also been suggested that decreased proliferative potential and early senescence of satellite cells is the primary cause of disease progression [3,39]. One group measured this limitation as an accelerated age-related shortening of satellite cell telomere length [40], a result contradicted by another study using a similar assay [41]. We observed no change in proliferative capacity of *mdx *satellite-derived cells. These findings suggest that the proliferative potential of satellite cells or their ability to self renew is not compromised; the number of satellite cells remains higher than controls throughout the lifespan of the animals ([32]; our observations).

In summary, our study is the first to provide data on the differentiation efficiency of satellite cells in older *mdx *and *PMP22 C22 *mice. We found that, generally, the age of the individual animal had little impact on the differentiation of satellite cells. The pathological processes in muscle of mice with *mdx *or *PMP22 C22 *genotype are not necessarily accompanied by defects in satellite cell differentiation. However, in some *mdx *individuals, satellite cell differentiation is impaired after the age of two months, in a manner akin to that we observed in *MSVski *hypertrophic muscle pathology [14]. It seems clear that there is heterogeneity in the pathology of muscle in various animal models of human disease, probably due to epigenetic/environmental contributions. It remains to be further investigated what causes cases of poor differentiation and whether this impaired differentiation of satellite cells enhances deterioration of the physical condition of the individual.

## List of abbreviations

BMD Becker type muscular dystrophy

CEE Chick embryo extract

CMT1A Charcot-Marie-Tooth disease type 1A

DAPI diamidino-2-phenylindole

DM differentiation medium

DMD Duchenne muscular dystrophy

DMEM Dulbecco's Modified Eagle's Medium

EDL Extensor digitorum longus

FCS fetal calf serum

FITC fluorescein isothiocyanate

HMSN1A Hereditary Motor and Sensory Neuropathy type1A

HS horse serum

IGF-1 insulin-like growth factor 1

MSV Master seed virus

MyHC Myosin heavy chain

mAb monoclonal antibody

PBS phosphate buffered saline

PFA paraformaldehyde

PM proliferation medium

PMP 22 Peripheral myelin protein 22

SD standard deviation

## Competing interests

The author(s) declare that they have no competing interests.

## Authors' contributions

MMS and CJM have contributed equally and have carried out the cellular experiments and drafted the manuscript. HB helped with the animals. CH provided *PMP22 C22 *animals. SMH conceived the study, participated in its design and coordination, and contributed to the writing. All authors read and approved the final manuscript.

## Pre-publication history

The pre-publication history for this paper can be accessed here:


